# Predictive histopathological factors of nodal metastasis in penile cancer

**DOI:** 10.1590/S1677-5538.IBJU.2022.0640

**Published:** 2023-05-30

**Authors:** Marcos Adriano Garcia Campos, Antonio Augusto Lima Teixeira, José de Ribamar Rodrigues Calixto, Joyce Santos Larges, Jaqueline Diniz Pinho, Gyl Eanes Barros Silva

**Affiliations:** 1 Universidade Estadual Paulista Faculdade de Medicina Botucatu SP Brasil Faculdade de Medicina da Universidade Estadual Paulista - Unesp, Botucatu, SP, Brasil; 2 Hospital Universitário Presidente Dutra Laboratório de Imunofluorescência e Microscopia Eletrônica São Luís MA Brasil Laboratório de Imunofluorescência e Microscopia Eletrônica, Hospital Universitário Presidente Dutra, São Luís, MA, Brasil; 3 Universidade de São Paulo Departamento de Genética Ribeirão Preto SP Brasil Departamento de Genética, Universidade de São Paulo, Ribeirão Preto, SP, Brasil; 4 Universidade Federal do Maranhão Departamento de Medicina II São Luís MA Brasil Departamento de Medicina II, Universidade Federal do Maranhão, São Luís, MA, Brasil; 5 Universidade Estadual do Maranhão Zé Doca MA Brasil Universidade Estadual do Maranhão, Zé Doca, MA, Brasil

## INTRODUCTION

Penile cancer (PC) is a neoplasm with variable incidence depending on geographic location, with higher prevalence in underdeveloped countries when compared with developed countries (1). Moreover, the poorest regions of Brazil have the highest incidence of PC, according to the literature (2–4). Because PC mainly affects a socially disadvantaged population from underdeveloped countries, knowledge of its pathology, clinical management, and treatment is still limited. However, it has been revealed that the presence and extent of inguinal lymph node metastasis are the most important prognostic factors of PC (5–7).

PC often metastasizes first to the superficial inguinal lymph nodes, then extends to the deeper nodes, and, finally, to the iliac lymph nodes (8). Enlarged lymph nodes >1.5cm in diameter, pathological stage T2 and above, low-to-middle differentiation, and lymphatic vascular infiltration were independent predictive factors that worsened the prognosis of patients with PC (9). Lymphadenectomy can interrupt this process and acts as a curative treatment for PC, avoiding radical procedures that may further impact quality of life and sexual function (10). Moreover, recent studies have revealed better survival outcomes in patients with microscopic metastases who undergo prophylactic inguinal lymphadenectomy (IL) compared with those whose physical examination initially showed no metastasis to the lymph nodes but who later had recurrent disease (11–15). Clinical and radiological assessments are insufficient to detect early lymph node metastasis. Therefore, prophylactic lymphadenectomy is a viable procedure for selected patients at high risk of metastasis, although, IL has a high rate of short- and long-term complications. Accordingly, several histological factors have been explored with regard to their potential to reliably predict the occurrence of metastasis in inguinal lymph nodes.

This study aimed to provide pathologists, oncologists, and urologists with a review of the main histopathological parameters that should be considered when deciding to perform lymphadenectomy, along with observations from the region with the highest incidence of PC worldwide (2).

### Histological type

Squamous cell carcinoma (SCC) constitutes 95% of PCs. The remaining 5% are classified as sarcoma (leiomyosarcoma, Kaposi's sarcoma, angiosarcoma, rhabdomyosarcoma, epithelioid sarcoma, and Ewing's sarcoma, melanoma, adenocarcinoma, or sebaceous carcinoma) (16). Although there is less evidence to support the use of lymphadenectomy in these other neoplasms, aggressive approaches should be considered in appropriate patients. Furthermore, it is important to consider tumor stage and nodal status when predicting the outcome of patients with non-SCC neoplasms (17).

In cases of penile melanoma, sentinel lymph node evaluation is similar to the established protocol for melanoma in other sites (18). Penile sebaceous carcinoma has a strong tendency to metastasize to regional lymph nodes; thus, it is usually treated with wide local excision and regional lymphadenectomy. Regional lymphadenectomy is performed only if clinically significant nodes are found. In a previous study, three of five patients with sebaceous carcinoma presented with bilateral palpable inguinal lymph nodes and underwent IL. In two of these three patients, a biopsy revealed nodal metastasis (19).

### Histological subtype

Penile SCC is classified into several subtypes that demonstrate varying rates of inguinal lymph node involvement and survival. The most recent World Health Organization classification divided SCC into two categories: human papillomavirus (HPV)-associated and non-HPV-associated SCC (20). Unlike in SCC of the head and neck, the presence of HPV in penile SCC does not necessarily dictate prognosis or therapeutic approach. Thus, classifying tumors as HPV+ or HPV- may not be as useful as grouping them into histological subtypes of low risk or high risk for developing lymph node metastasis.

The frequency of SCC subtypes varies according to geographic location. In northeastern Brazil, HPV was detected in approximately 89.1% of pe-nile SCC cases (21), a rate higher than that observed in other regions, which have a prevalence of 1.3–72.9%. In our previous study, we showed many HPV-associated subtypes, differing from other regions, characterized by a large predominance of the usual variant (HPV−) (22).

Given the importance of the SCC histological subtype, it is vital that adequate tissue representation is used in macroscopy for the correct subclassification of these tumors. This is especially true for regions of high incidence, where patients seek medical assistance at a very advanced stage, with an average of almost 2 years after the first signs of the disease and large tumors measuring approximately 4.5 cm (3). In these cases, more than one pattern is often observed macro and microscopically, and each of them may have different degrees of differentiation and a different prognostic profile, considering the high frequency of mixed subtypes in our cases, particularly in advanced tumors. Given these peculiarities regarding morphological criteria, we recommend that the same professional should conduct all diagnostic steps, from macroscopy to microscopy. In the next section, we group the subtypes based on the risk of developing lymph node metastasis.

### Low-risk group

In this section, we group the PC subtypes according to the risk of developing lymph node metastasis. Verruciform neoplasms constitute one class of PC that is at low risk of metastasis, regardless of the presence or absence of HPV. The prototype of this exophytic pattern is verrucous carcinoma, an HPV− *in-situ* neoplasm that is rarely invasive. Therefore, *in-situ* tumors and verrucous SCC are not recommended for IL, even when there is clinical suspicion of nodal involvement. In fact, there are no reports of metastasis in patients with these tumors. Usually, antibiotic treatment is initiated for enlarged nodules, and the nodule is excised if enlargement persists (23). Other subtypes of verruciform carcinoma that are not associated with HPV and exhibit low rates of lymph node metastasis include pseudohyperplastic, papillary, and cuniculatum SCC.

Condylomatous carcinoma is a form of verruciform carcinoma that is associated with HPV and demonstrates a low rate of inguinal metastasis of approximately 17% (24). Nevertheless, more advanced tumors with a higher level of infiltration are more likely to lead to lymph node metastasis. In our country, where the neoplasm is diagnosed after 2 years of disease progression, the lesions are large, often forming part of a mixed-pattern neoplasm, especially with the usual type. In these cases, the patient faces a less favorable prognosis, usually with a more aggressive carcinoma component.

### High-risk group

We classified tumors as high-risk if they demonstrate a risk of lymph node metastasis at a diagnosis rate of > 50%. Like low-risk tumors, there are representatives of both HPV+ and HPV− tumors in this category. Non-HPV-associated subtypes include the usual (pattern solid), pseudoglandular and sarcomatoid, showing a risk of nodal involvement above 85% (25).

Among the subtypes associated with HPV are basaloid, clear cell, and mixed forms of SCC such as warty-basaloid. The risk of lymph node metastasis in these subtypes ranges from 50% to 66% in basaloid SCC to 100% in clear cell SCC (26). Other HPV+ forms of PC, such as lymphoepithelioma-like and medullary cancer, are high-grade neoplasms rich in inflammatory cells, but their prognosis has not yet been established.

Hybrid, warty-basaloid, and papillary-basaloid carcinomas should be further evaluated according to the percentage of the highest risk component. Thus, the tissues of the lesions should be accurately represented and observed to detect different subtypes. At our institution, lesions are well represented, with those smaller than 3.0 cm being fully represented, and the larger lesions being prepared with at least 30 blocks of paraffin.

### Histological grade

Tumor histological grade is the most important prognostic factor in PC patients with clinically negative lymph nodes that do not undergo regional lymphadenectomy (27). The National Comprehensive Cancer Network (NCCN) and the European Association of Urology (EAU) have published guidelines on the management of PC based on the histological grade and staging of the primary tumor (pTNM, AJCC). In epithelial tumors, it is generally more difficult to define the histological grade of squamous carcinomas than in adenocarcinomas. Moreover, classification criteria vary according to the institution, resulting in high interobserver variability in the grading of PC (28). Additionally, it is important to note that knowledge of SCC in other sites does not necessarily apply to SCC of the penis.

The morphological features commonly used to assess SCC grade are keratinization; cell atypia/anaplasia calculated by the nucleus to cytoplasm ratio; thickness of the cell membrane; nuclear pleo-morphism and chromatin pattern; pattern of tumor growth and expansion in nests, cords, solid blocks, and detached cells; and presence of nucleolus, mitotic activity, intercellular bridges, and tumor edge (29). Tumor grading is classified as follows: G1: well--differentiated, tumors with minimal changes and morphological proximity to a normal or hyperplastic epithelium, and atypia in the most basal layer; G2: moderately differentiated, tumors with alterations between G1 and G3; and G3: poorly differentiated, tumors with any percentage of cell anaplasia (8). This classification system demonstrates the importance of accurate sampling to detect small areas of undifferentiated cells.

The risk of nodal involvement, as well as tumor invasiveness and aggressiveness increase with histological grade. Specifically, nodal metastasis is found in approximately 8%, 50%, 60% of G1, G2 and G3 tumors respectively (30, 31).

### Tumor location and measurement

The glans is the most frequent site of involvement in PC, followed by the foreskin. Tumors of the foreskin have a better prognosis than those of the glans because they are of a lower grade and are more superficial, thus demonstrating less potential for nodal metastasis.

Although tumor size is not a good predictive factor for penile SCC, tumors 2–4 cm in size are more likely to be associated with nodal metastasis, in contrast to tumors smaller than 2 cm or larger than 4 cm. This is due to tumors with superficial dissemination (verruciforms) that reach large proportions (32, 33).

It is important to determine advanced loco--regional disease to define its management. Primary radical inguinal surgical debulking alone for these cases is unlikely to promote long-term survival and is related to a high incidence of complications (34)

### Presence of koilocytosis/HPV

Koilocytosis is a morphological parameter indicative of the presence of HPV that should be included in the histopathological report. Through polymerase chain reaction, HPV has been identified as an important prognostic biomarker for penile neoplasia because of its tumorigenic pathway in SCC and its occurrence in tumor tissues (35). At least two Brazilian studies have identified an association between koilocytosis and a low incidence of lymph node metastasis (36, 37). However, further research is warranted for confirmation.

### Perineural invasion

Perineural invasion is characterized by infiltration of the clear space surrounding the nerve bundle under the epineurium and should not be confused with the nerve trapped within the tumor mass. The role of perineural invasion in PC is controversial. Some researchers have declared the presence of perineural invasion to be associated with a high risk of inguinal lymph node metastasis in PC patients (30, 38). Others, including studies from Brazil, have found different results (36, 38–40). In 2009, the EAU guidelines identified perineural invasion as an important prognostic factor in lymph node metastasis (41), although the same recognition was not given to the 2014 EAU, 2017 NCCN, or the eighth edition of the AJCC TNM staging guidelines.

### Lesion depth/tumor thickness

The depth of invasion and tumor thickness are often confused, and although they represent different measurements, they have equivalent significance. The depth of invasion is measured from the intact basement membrane of the tumor edge to the deepest tumor cell. Tumor thickness, in turn, is measured from the top of the neoplasm to the deepest tumor cell. In exophytic and keratinizing lesions, tumor thickness is measured from the surface, excluding the keratin layer; in ulcerated lesions, tumor thickness is measured from the surface of the ulcer (3, 6, 32).

The mean thickness of neoplasia-free penile tissue to the lamina propria is 3 mm (T1), to the corpus spongiosum is 5 mm (T2), and to the corpus cavernosum is 10 mm (T3). In the foreskin, the thickness from the skin to the mucosa is approximately 10 mm (30).

Studies have shown a correlation between tumor thickness and lymph node metastasis index. Additionally, higher tumor infiltration and histological grade are correlated with a greater likelihood of lymph node metastasis. Thus, tumors with a thickness of <5 mm have a minimal risk of metastasis, those with a thickness of 5–10 mm have an intermediate risk of metastasis, and those with a thickness of >10 mm have a high risk of metastasis (approximately 80–86%). Nevertheless, due to anatomical variation in thickness, we believe TNM staging classification (based on anatomical structure) to be more efficient in assessing of depth of invasion than the measurement in millimeters (11).

### Growth pattern and invasion front

Neoplasm growth patterns can be horizontal or vertical and correspond to the form of tumor spread and the relationship with the host tissue. Some studies have determined the vertical growth pattern to be associated with a more unfavorable prognosis compared to the horizontal growth pattern (42, 43). Moreover, the horizontal growth pattern is typically found in exophytic verruciform tumors.

Recently, researchers have turned their attention to the assessment of the so-called “invasion front” (44). Translocation of neoplastic cells is a well-known feature at the invasion front of malignant tumors. The change in the phenotypic pattern of invasion with the absence of epithelial biomarkers and the presence of mesenchymal biomarkers may be associated with invasion and lymph node involvement (45, 46). Unlike in colorectal and head and neck tumors, no studies have yet assessed tumor budding in PC. Thus, future study of this topic is warranted.

### Nomograms

Several nomograms have been created to predict lymph node metastasis (29, 47, 48). However, the diverging importance of each individual histological parameter results in poor performance of the combination of these parameters in nomograms. Another challenge of using these nomograms is the lack of independent external verification and validation. Nomograms applied by different groups to the same population did not obtain the same results (49, 50).

### Ki67, p53, and p16

Ki67, p53, and p16 have been evaluated as potential biomarkers of prognosis and lymph node metastasis in PC (51). Although p53 demonstrated the best predictive ability among the three biomarkers, it was not shown to be better than that of other predictive factors, such as tumor stage, and there are no consistent results concerning its use in the ma nagement of PC (52). Moreover, Brazilian studies have found good results with the evaluation of p53 (53, 54). Furthermore, a strong association between high Ki67 expression and lymph node metastasis in PC has been reported (55, 56). Other studies have confirmed this association (57, 58). Although the absence of p16 may be associated with poor survival, most studies did not find an association between p16 and lymph node metastasis (21, 59, 60).

**Figure 1:**
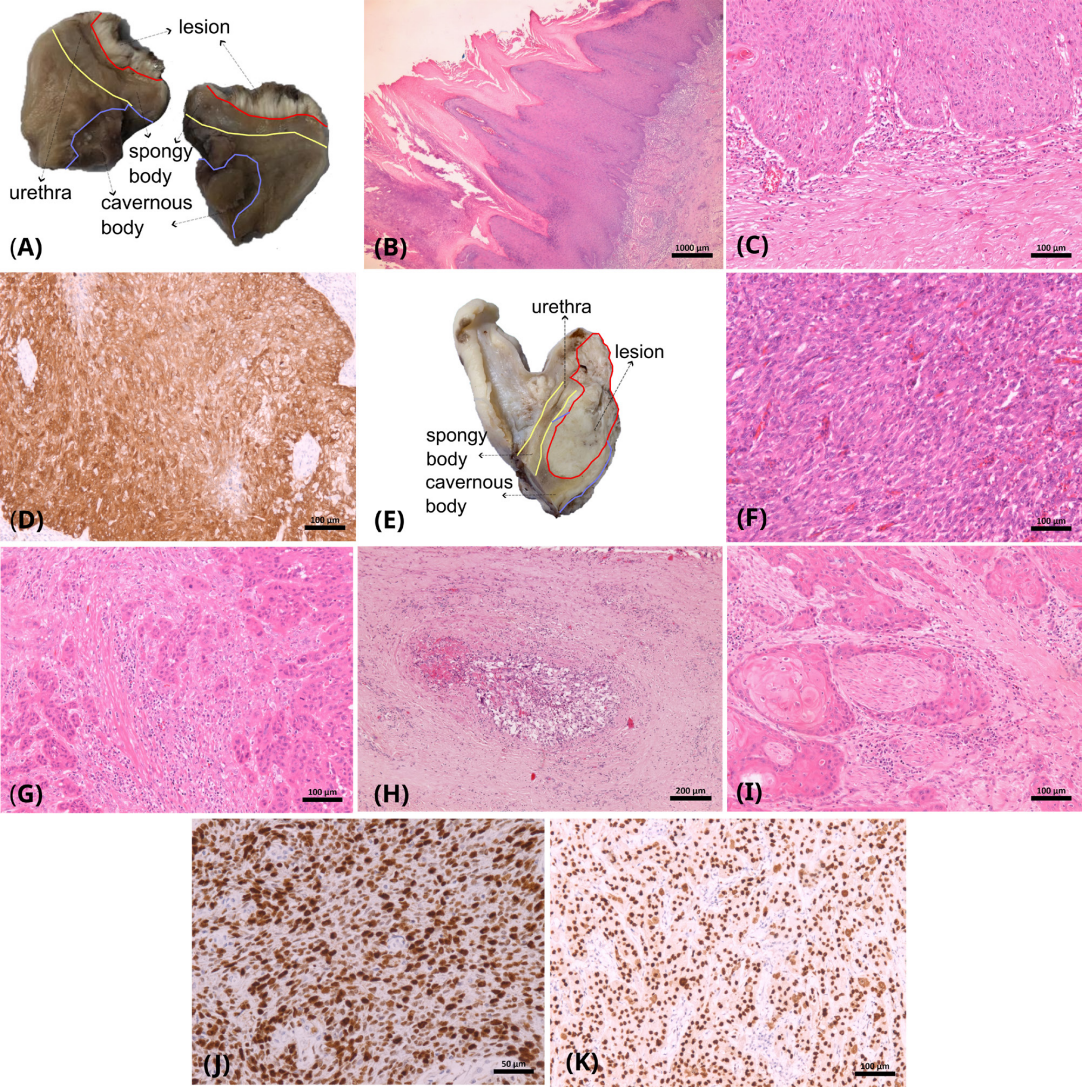
Pathological features associated with low risk (A, B, C, D) and high risk (F, G, H, Y, Z) lymph node metastases.

Immunohistochemical biomarkers require further investigation as they are simple to employ and are widely used. Moreover, in Brazil, the use of immunohistochemical biomarkers is funded by the public health system. Finally, it is noteworthy that the three biomarkers listed above can be assessed in any basic pathology laboratory. Our group will soon present the results of a study using these biomarkers.

## CONCLUSIONS

No definitive predictive biomarker of inguinal lymph node metastasis has yet been established. There are many challenges to achieving this goal: the disease is most prevalent in regions with low socioeconomic conditions, there is difficulty in standardizing the criteria for inguinal lymph node metastasis, there is difficulty in accessing radiological exams and medical monitoring of patients, there is varying prevalence of HPV and histological subtypes according to geographic location, there is interobserver variation, and there is the need for extensive tissue sampling in advanced tumors. However, several studies and international guidelines demonstrate that the strongest predictors of inguinal lymph node metastasis are the stage of the primary tumor, the histological grade, and the presence of angiolymphatic invasion.
